# Eye donation – Awareness and willingness among attendants of patients at various clinics in Melaka, Malaysia

**DOI:** 10.4103/0301-4738.73727

**Published:** 2011

**Authors:** Sulatha Bhandary, Rajesh Khanna, Krishna A Rao, Lavanya G Rao, Kamala D Lingam, V Binu

**Affiliations:** Department of Ophthalmology, Kasturba Medical College, Manipal, Karnataka, India; 1 Department of Statistics, Kasturba Medical College, Manipal, Karnataka, India; 2 Department of Ophthalmology, Melaka Manipal Medical College, Melaka, Malaysia

**Keywords:** Awareness, eye donation, willingness, Malaysia

## Abstract

**Aim:**

Corneal blindness accounts for 3.42% of blindness in Malaysia; the rate of eye donation is low. The aim of the study was to assess the awareness about eye donation and willingness to donate eyes among attendants of patients at various clinics in Melaka, Malaysia.

**Materials and Methods:**

This observational study was conducted on attendants who accompanied patients (*n* = 400) visiting various outpatient departments of the General Hospital and two peripheral clinics in Melaka between August and October 2007. The participants answered a questionnaire (Malay and English versions) which included demographic profile, awareness of eye donation, knowledge regarding facts of eye donation, and willingness to donate eyes. Univariate and multivariate logistic regression was performed at 5% level of significance.

**Results:**

Awareness of eye donation was observed in 276 (69%) participants. Multivariate analysis showed that awareness was more among females when compared to males (*P* = 0.009). Of the 276 participants who were aware of eye donation, only 34.42% were willing to donate eyes. Willingness was more among the Indian race (*P* = 0.02) and males (*P* = 0.02). Educational status did not influence the willingness to donate eyes.

**Conclusions:**

Although majority of participants were aware of eye donation, willingness to donate eyes was poor.

Corneal blindness accounts for about 6–8 millions of blind persons in the world.[1] In Malaysia, it accounts for 3.42% of all blindness.[2] Corneal transplantation offers the potential for sight restoration; however, it is hugely dependant on voluntary eye donation by suitable donors.[1]

A review of the literature using PUBMED revealed awareness studies among nonmedical faculties, and university students in Malaysia, but not among the general population.[3,4] The aim of the study was to assess the awareness of eye donation and willingness to donate eyes among the attendants of patients at various clinics in Melaka, Malaysia.

## Materials and Methods

The study was conducted among the attendants of patients (relatives or friends) visiting outpatient departments of the General Hospital and two peripheral clinics in Melaka, Malaysia. The study period was from August 2007 to October 2007. Anticipating a prevalence of 80% awareness, 4% absolute precision, and 95% confidence level, the minimum sample size required was 384.[5]

### Data collection

The population of Melaka state is about 455,300.[6] The General Hospital in Melaka caters to a population of about 500,000 including patients from neighboring states, with a rough total of 1000 outpatients per day in all the outpatient departments. The two peripheral clinics see roughly about 300 outpatients per day. Based on the sample size calculation, a total of 400 participants were recruited by a convenient sampling method. The attendants waiting outside various outpatient departments of the above-mentioned hospital and clinics, other than Department of Ophthalmology, were included in the study. After written informed consent, the participants were asked to answer a questionnaire either in English or Malay. The Malay questionnaire was verified by translating it back to English to ensure that there was no difference between the Malay and the English version. The questionnaire (appendix) included questions pertaining to demographic profile, awareness about eye donation and willingness to donate eyes. “Awareness” was considered as having a realization about the fact that a dead individual’s eyes can be utilized to give vision to those blind from corneal disease. “Knowledge” was considered as having understanding regarding the details of different aspects of eye donation like pledging of eyes, who can donate eyes, when can eyes be donated, and what steps are to be taken after death to donate one’s eyes. Literate subjects self-administered the questionnaire; the chief author read out the questionnaire to the participants with no formal education. Those subjects not willing to participate in the study were excluded (*n* = 30). The occupation of the participants was categorized as paramedical and nonmedical, with an understanding that individuals in paramedical occupations are likely to have a better awareness compared to nonmedical personnel. Paramedical occupation included nurses, ward boys, and helpers working in various hospitals, while nonmedical profession included all other occupations not related to the medical field. Data were entered and analyzed using the “Statistical Package for Social Sciences” (SPSS) version 15.0 (SPSS Inc., Chicago, IL, USA). Univariate and multivariate logistic regression was performed to find the variables associated with awareness and willingness. Statistical significance was determined at 5%.

## Results

Malaysia being a multiracial country, a total of 400 subjects of Malay, Chinese, and Indian races participated in the study. The mean age of participants was 37.93 years (standard deviation [SD] 15.1 years) with a range of 18–75 years. A slight female preponderance of 55.7% was seen. The ethnic distribution was 269 (67.3%) Malays, 77 (19.3%) Chinese, and 54 (13.5%) Indians. Fifty-three subjects (13.2%) had either no formal education or primary education only, 245 (61.3%) had secondary education, and 102 (25.5%) were graduates or postgraduates.

The awareness of eye donation was found to be 69% (*n* = 276). Univariate analysis [Table 1] showed that individuals with a higher education level (graduate and above), the younger age group (18–45 years), individuals of paramedical occupation, and females were more aware of eye donation (*P* < 0.05). However, on multivariate analysis [Table 2], only the female gender was significantly associated with the awareness of eye donation (*P* = 0.009).

**Table 1:** Univariate analysis showing an association between awareness of eye donation and various variables (*N* = 400)

Variable	Aware (%)	Odds ratio (95% CI)	*P*-value
Age (years)			
18–45 (*n* = 252)	183 (72.6)	1.6 (1.02, 2.45)	0.04*
46–80 (*n* = 148)	93 (62.8)	1	
Gender			
Male (*n*= 177)	108 (61.0)	1	0.002*
Female (*n* = 233)	168 (75.3)	1.95 (1.27, 3)	
Race			
Malay (*n* = 269)	188 (69.9)	1	0.3
Chinese (*n* = 77)	49 (63.6)	0.75 (0.44, 1.28)	0.73
Indian (*n* = 54)	39 (72.2)	1.12 (0.59, 2.15)	
Education status			
Primary/no formal education (*n* = 53)	36 (67.9)	1	0.56
Secondary education (*n* = 245)	156 (63.7)	0.83 (0.44, 1.56)	0.044*
Graduates and above (*n* = 102)	84 (82.4)	2.2 (1.02, 4.76)	
Occupation			
Paramedical (*n* = 46)	38 (82.6)	2.32 (1.05, 5.12)	0.038*
Nonmedical (*n* = 354)	238 (67.2)	1	

* Indicates statistically significant

**Table 2:** Multivariate logistic regression showing the relation between awareness and willingness for eye donation with various variables

Variables	Awareness	*P*-value	Willingness	*P*-value
	Odds ratio and 95% CI		Odds ratio and 95% CI	
Age (years)				
18–45	1.26 (0.78, 2.02)	0.35	1.06 (0.59, 1.89)	0.86
46–80	1		1	
Sex				
Male	1	0.009*	1	0.02*
Female	1.82 (1.16, 2.85)		0.53 (0.31, 0.9)	
Race				
Malay	1	0.73	1	0.23
Chinese	0.91 (0.52, 1.58)	0.38	1.51 (0.77, 2.95)	0.02*
Indian	1.36 (0.69, 2.68)		2.37 (1.14, 4.94)	
Education				
Primary/no formal education	1	0.44	1	0.39
Secondary	0.77 (0.40, 1.49)	0.09*	0.71 (0.33, 1.55)	0.68
Graduates and above	2.0 (0.89, 4.48)		0.84 (0.36, 1.96)	
Occupation				
Others	1	0.09*	1	0.3
Paramedical	0.49 (0.22–1.13)		0.64 (0.28, 1.47)	

* Indicates statistically significant

Of the 276 participants, who were aware of eye donation, 95 (34.42%) were willing to donate eyes. Univariate analysis showed that Indian race and males were more in favor of donating eyes (*P* < 0.05) [Table 3]. Multivariate analysis [Table 2] confirmed that gender and race are independent factors contributing to willingness to donate eyes.

**Table 3:** Univariate analysis showing association between willingness for eye donation and various variables (*N* = 276)

Variable	Willing (%)	Odds ratio (95% CI)	*P*-value
Age (years)			
18–45 (*n* = 183)	59 (32.2)	0.75 (0.45, 1.27)	0.29
46–80 (*n* = 93)	36 (38.7)	1	
Gender			
Male (*n* = 108)	48 (44.4)	1	0.005*
Female (*n* = 168)	47 (28)	0.49 (0.29, 0.81)	
Race			
Malay (*n* = 188)	55 (29.3)	1	0.123
Chinese (*n* = 49)	20 (40.8)	1.67 (0.87, 3.2)	0.009*
Indian (*n* = 39)	20 (51.3)	2.55 (1.26, 5.14)	
Education status			
Primary/no formal education (*n* = 36)	15 (41.7)	1	0.274
Secondary education (*n* = 156)	50 (32.1)	0.66 (0.31, 1.39)	0.54
Graduates and above (*n* = 84)	30 (35.7)	0.78 (0.35, 1.73)	
Occupation			
Paramedical (*n* = 38)	9 (23.7)	0.55 (0.25, 1.21)	0.14
Nonmedical (*n* = 238)	86 (36.1)	1	

* Indicates statistically significant

Among those who were aware (*n* = 276) about eye donation, 88% knew that eyes could be donated only after death. No disfigurement of the face as a result of eye donation was documented by 76.2% of participants. Anonymity about the donor and recipient was known to 74.5% of participants. The most important source of awareness was the media (55.4%) with newspapers topping the list (36.7%). The fact that the next of the kin had the right to give consent was known to 55.6%, but 36.2% were not aware that the eyes could be preserved after retrieval. The following were misconceptions: 90.3% of the participants thought that eyes could not be retrieved at the house of the deceased, and 43.3% felt that the whole eyeball was transplanted to the patient.

There was no statistically significant association between most questions (appendix) regarding the knowledge of eye donation and race; however, 31% of Indians reported that eye donation was against their religion while the corresponding percentages reported by Chinese and Malays were only 12% (*P* = 0.011).

## Discussion

In the present study, 69% of the participants were aware of eye donation; the Chinese population was less aware of eye donation compared to Malays and Indians. In contrast, a study by Yew *et al*. showed a better awareness (80.7%) among Singaporeans, and Chinese were more aware of eye donation when compared to Malays and other races.[5]

The region-specific factors may influence the knowledge of eye donation in different parts of the world. A study by Priyadarshini *et al*. showed an awareness of 50.69% among the patients attending two outreach clinics in southern India.[7] However, the awareness was only 30.7% in the rural population of south India, compared to 73.8% in the urban population of India.[8,9] A study by Tandon *et al*. in Delhi found that 55.4% next of kin were aware of the concept of eye donation.[10]

Although 69% of the participants had the awareness about eye donation, the willingness to donate eyes was seen in only 34.42%. This finding of better awareness than willingness to donate eyes is well observed in the study by Yew *et al*. in Singapore (awareness 80.7% and willingness 67%).[5] Also, the study by Tandon showed that the prior knowledge of eye donation, literacy, and socioeconomic status had no influence on willingness for eye donation and major reasons for not donating eyes included refusal to discuss the issue and dissuasion by distant relatives, legal problems, and religious beliefs.[10]

In our study, Indians were more willing to donate eyes compared to Malays and Chinese, although our study showed that 31% of the Indians felt eye donation is against their religion while only 12% of Malays and Chinese shared the same opinion. Many studies have shown that ethnicity has an important role in the willingness of organ donation.[11–13] Probably, the reasons for the unwillingness could have been culture-specific issues arguing against donation including a sense of the sacredness of the body, belief that it is important to have an intact body after passing away and fear of illegal trade in organs and the poor would suffer.[5,11,14]

In the present study, 84.5% were aware that eye donation is not against their religion and 76.2% knew that it does not cause any disfigurement of the face. In contrast, other studies have shown a concern among the respondents, like disfigurement of the face of the deceased, fear that it is against their religious belief, and may be time-consuming thus delaying the funeral process.[15]

Educational status showed a positive impact on the awareness of eye donation but did not show any statistically significant effect on the willingness to donate eyes. However, Yew *et al*. have found that the knowledge and willingness is more due to the higher educational status in Singaporeans.[6] According to a study by Shahbazian *et al*., “age, sex, and occupation did not influence the attitudes; however ethnicity, educational level, economic status, and having a loved one in need of an organ significantly increased the willingness for organ donation.”[12]

In our study, although females were more aware of eye donation, similar to the observations by Krishnaiah *et al*., they were reluctant to donate eyes.[8] Unwillingness among females may be because of their family ties and the necessity to seek permission from the family members before pledging their eyes. Hayward *et al*. have shown that women were more concerned about the transmission of diseases or of personality following organ donation.[16] The older age group was more willing to donate eyes compared to the younger age group again consistent with the Indian study.[8]

According to the National Eye Survey 1996, corneal pathology accounts for 15.1% of the childhood blindness in Malaysia.[2] The majority of the donor corneas were imported from the USA (*n* = 112 or 71%) and Sri Lanka (*n* = 27 or 17%).[17] The eye donation among the local ethnic groups was a total of 19, of whom 8 were Chinese (42%), 6 Indians (32%), and 4 were Malays (21%) in year 2005 and data was not available in one case.[17] In year 2004, none of the Malays had donated eyes and the new transplant rate was 7 per million populations in 2004.[17] This clearly indicates though there is a great demand of donor eyes, the donation in the community is very less. Studies show that there was poor awareness about the “Fatwa” regarding organ donation, passed by the Muslim law council in 1995.[15,18] This lack of awareness has led to the fear of doing something against religion by donating organs among the population.[15,18]

Thus, to make this dream of reducing the burden of avoidable corneal blindness, the ophthalmologists, general physicians, nongovernmental organizations (NGOs), and especially the religious leaders have to work in unison to educate and motivate people to donate eyes.

### Limitations of the study

The limitation of this study was that the study participants were attendants of patients in a hospital and peripheral clinics so the results of this study cannot be generalized to the Malaysian population. A population-based house-to-house survey would have been ideal.

Although majority of participants were aware of eye donation, only one-third (34.42%) of them were willing to donate their eyes. The majority of Malays were not willing to donate eyes although they agree that eye donation is not against their religion. Therefore, there is a great need to have awareness programs to motivate people to donate eyes to help mankind by giving sight to the needy.

## Appendix (Questionnaire)

**Table:**

Screening of awareness of eye donation					
1. Name:____________________________________________________					
2. Age__________ years__________					
3. Sex:	Male □	Female □			
4. Race:	Malays □	Chinese □	Indian □		
5. Occupation:	Paramedical □	Nonmedical □			
6. Address:____________________________________________________					
7. Education:	Primary or no formal education □		Secondary □		
	Graduate and postgraduate □				
**Questionnaire:**					
1. Can eyes be donated?				Yes □	No □
If **YES**, answer the following-					
2. Eye donation means-					
Service to mankind □			giving sight to blind □		
Donation of eyes after one’s death. □			Do not know □		
3. One can donate our eyes only after death.				Yes □	No □
4. Ideally time duration to retrieve eyes is within 6 hours after death.				Yes □	No □
5. The next of kin (first degree relative) has the right to give the consent for eye donation.				Yes □	No □
6. A person with communicable disease can donate his eyes.				Yes □	No □
7. Regarding transplantation – Only the cornea (layer in front of the black portion of the eye) is used for the transplantation. □ Or					
The whole eyeball is transplanted. □					
8. Eye donation disfigures the face of the donor.				Yes □	No □
9. The eyes can be removed at donor’s house itself.				Yes □	No □
10. The name of the donor and recipient remains anonymous.				Yes □	No □
11. The donor eyes can be preserved in the eye bank.				Yes □	No □
12. Eye donation is against your religious belief.				Yes □	No □
13. Are you willing to donate your eyes?				Yes □	No □
14. Source of awareness:	Family physician □		Family members □	Friend □	
	(Media) newspaper □		television □	Internet □.	
